# On the Reputed Anti-Epileptic Power of Argentum Nitratum

**Published:** 1801-05

**Authors:** R. Kinglake

**Affiliations:** Chilton, super Poldon


### *On the reputed Anti-epileptic Power of Argentum Nitratum.*

To the EDITORS of the MEDICAL and PHYSICAL JOURNAL.

Gentlemen,

IF you fhould coincide with me in opinion, that medical fcience is not lefs advantageoufly promoted by faithfully recording inftances of unfuccefsful practice, than by anxioufly detailing fuccefsful cafes, you will, probably, deem the fubfequent narrative of the infufficiency of the nitrate of filver, (Argentum Nitratum) in the cure of Epilepfy, not unworthy of public notice.

 The

The reputation which this chemical compound has acquired for its falutary influence in fubduing this difeafe, refts on the authority of confiderable experience, and has been recently corroborated by communications in your Journal, from Drs. Cappe and Boftock,* men whofe acquaintance I highly value, and whofe judgement and veracity claim my utmoft deference. Warranted by fuch credible evidence in its favour, I lately entered on its ufe in two cafes of epileptic affection, with more than my accuftomed confidence in medicinal efficiency, even when oppofed to lefs formidable difficulties.

The firft cafe in which its employ came under my direction, was that of an elderly woman, of a weakly temperament, but not vitiated by any habitual diforder, who had been afflicted with epileptic attacks at irregular intervals, during about eighteen months; they ufually recurred once a week, occafionally oftener, and a refpite of ten days had been very rarely experienced. Neither the violence nor duration of the paroxyfms offered any thing remarkable; nor were the fubfequent inconveniences in any refpect extraordinary.

This patient began with half a grain of the nitrate of filver, formed into a pill with crumb of bread, and was ordered to repeat it at equal diftances, three times a day; each dofe during the firft day, induced afflicting ficknefs, without the alleviation of vomiting. Difcouraged by this unexpected occurrence, the patient could only be perfuaded to profecute the medicine, by being affured that it was accidental, and not due to its ordinary operation. Sufpecting that fome inaccuracy had happened in difpenfing the prefcription, enquiry was made, when it appeared, that in the abfence of the apothecary to whom it was fent, a perfon not fufficiently converfant with medicinal compofition, had probably exceeded the quantity directed. The utmoft exactnefs was then obferved in preparing the pills, which were taken as before, but without any fenfible effect: No variation was made in the dofe for three days; and no influence, during that time, was manifefted by the medicine. It was then augmented to one grain three times a day; this was continued a week, in the courfe of which, a paroxyfm, as ufual, occurred, and no impreffion was felt from the medicine. It was now increafed to two grains at fimilar intervals, and perfifted in upwards of ten days; flight ficknefs was occafionally experienced, but no difference was induced either in the periods of recurrence, or the violence of the epileptic fits. The dofe was ultimately extended to three grains, at the diftances before ftated, and

* See Medical and Phyfical Journal, Vol. I. pages 184, 222.

and profecuted about three weeks, without any other proof of efficiency, than that of tranfient heat in the ftomach, and flight naufea foon after taking it.

It will appear from the preceding ftatement, that the nitrate of filver was in this cafe freely exhibited, and its employ fufficiently protracted for its full effect to have been produced; its complete failure, therefore, authorifes the conclufion, that in this inftance of epileptic affection it was wholly deftitute of curative power.

The fecond epileptic cafe in which the nitrate of filver was put to the teft of experiment by my advice, was that of a young man, about thirty years of age, of a robuft temperament, and free from any other habitual difeafe: The diforder commenced in his infancy, and accompanied every ftage of his growth to the ftate of manhood, with but little variation either in the violence, frequency, or duration of the paroxyfms. It was confidered as a rare occurrence for a week to elapfe without an attack; feveral ufually happened during that period. The difeafe feemed at length to have acquired an harmlefs familiarity with the general health, and the intervals of its recurrence were not ruffled by any fenfible degree of morbid derangement.

As may be fuppofed in the courfe of fo long a period as twenty-five years, every chance of relief was afforded, which either reflection or accident could devife or fupply; no lafting benefit, however, had been obtained. Although the duration of the difeafe prefented every difficulty which an eftablifhed affociation of morbid movements could engender, the apparently unimpaired ftate of the general health induced me to fuppofe that no irremediable organic læfion had been produced. Under this perfuafion, without holding out the poffibility of fpeedy aid, or of even promifing ultimate benefit, I judged it expedient to afford the patient a full chance of any advantage that might refult from the fucceffive employ (if neceffary) of various efficient medicines. Purfuant to this defign, digitalis, opium, cuprum vitriolatum, zincum calcinatum, acidum fulphuricum, argentum nitratum, hyofcyamus niger, and cicuta, were feverally directed; the dofes, intervals of adminiftration, length of continuance, and refults of each of which, were as here fpecified. Digitalis was taken from one grain to five, by flow augmentation three times a day, for fix weeks, during which time occafional giddinefs was experienced, but without naufea, or difcoverable retardation of pulfe; the intervals of the paroxyfms were lengthened to about ten days, and the attacks were lefs violent.

Opium was taken from one grain to three, by gradual increafe creafe three times a day, during a fortnight, in which time but one flight fit recurred; but the pulfe was rendered unufually full and flow, and an irrefiftible propenfity to fleep, with a threatening dilatation of the pupils enfued.

The digitalis and opium were now directed in conjunction, in dofes of two grains each, three times a day, and continued for two months, during which time two paroxyfms only returned; but this extraordinary fufpenfion feemed to have been purchafed at the expence of an alarming degree of vertiginous affection.

The cuprum vitriolatum was taken from one grain to five, during three weeks, three times a day, with the effect of inducing occafional naufea, and an herpetic determination on the cuticular furface; but no perceptible variation in the epileptic paroxyfms.

The zincum calcinatum was taken from five to fifteen grains, three times a day, for three weeks, without any obfervable influence on the difeafe.

The acidum fulphuricum was taken from thirty drops to two drachms, largely diluted with water, three times a day during one month, with the effect of fhifting the period of recurrence a few days later than the previous time of acceffion; but it may be remarked, that the patient, judging from his improved feelings of perfonal ftrength and mental energy, expreffed more confidence in the curative power of this medicine than in any thing which had been before adminiftered.

The argentum nitratum was now taken, from half a grain to four grains three times a day for one month, without difcovering the *smalleft influence*, either in arrefting or modifying the epileptic paroxyfms, and with no other effect than that of inducing more or lefs durable fenfation of burning heat in the ftomach.

The hyofcyamus niger was taken in fubftance from one grain to ten, three times a day during fix weeks, in which time but two paroxyfms recurred, and thefe with diminifhed violence and duration; but in the laft week of its employ, a degree of vertiginous ftupor fupervened, which feemed to indicate that the progreffive agency of this medicine was more likely to fuperinduce a dangerous malady than radically to alter the unknown diftempered conditions of vital motion, that form the proximate caufe of epilepfy.

The cicuta was given in the form of infpiffated juice (fuccus cicutæ fpiffatus), from five grains to fifteen, three times a day for one month; latterly it induced temporary giddinefs, but the paroxyfms returned with increafed frequency and violence during its employ.

The

The purpose of entering into this detail of the various effects of the different substances employed, is to exhibit a comparative view of the inefficiency of the argentum nitratum. The cuprum vitriolatum, zincum calcinatum and cicuta were its congeners in inertness, or at least complete insufficiency for affecting any salutary change in the disease. It may be objected, that two cases do not afford adequate experience for determining the powers of a medicinal agent; this must be admitted, but its force will be obviated by my offering them as a *contribution* only to the more extensive evidence of similar inefficacy adduced by the ingenious and accurate Dr. Magennis, in a late number of your Journal.* In that communication is recorded testimony on this subject, that demonstrates a diligence of research and tenaciousness of truth but rarely combined, yet essentially requisite to the practical investigation of medicinal power.

In having my opinion biassed by actual observation, and the admissible authority cited against the reputed efficacy of argentum nitratum in the cure of epileptic affection, it is not my wish to deny it the possibility of proving remedial under certain constitutional circumstances (not yet explained) favourable to its salutary operation; but this qualification divests it of all pretension to invariable effect, or to any influence that approaches to the character of specific power, and reduces it to the standard of all other efficient medicinal agents, that of occasionally inducing changes in the morbid actions of animal life that lead to the regeneration of health.

It would be important to distinguish what temperamental, morbid, or accidental circumstances are most conducive to the curative influence of argentum nitratum in this disease; until this information be furnished, the experience under this consideration authorises the expectation, that the indiscriminate employ of this agent in epilepsy will be more precarious in its effects, than may be presumed from the use of several other medicinal substances of acknowledged power in certain diseases, such as the cinchona in intermittent fever, mercury in syphilis, opium in morbid excess of sensibility, digitalis in pulmonary consumption, &c. Were, indeed, as much ascertained of the most beneficially operative powers of argentum nitratum as of these enumerated, the respective incertitude of effect common to them all would yet attach to it, and render it at best a medicine of doubtful agency.

It will not be alleged, that uncertainty of operation is inapplicable to the most efficacious articles of the Materia Medica. It

* See the Medical and Physical Journal, No. XXI. page 417.

It will be recollected, that organic susceptibility for medicinal impressions is, even *constitutionally*, widely various, and, *habitually*, infinitely different. On this diversity, then, rests the greater or less discordancy of influence from similar agents.— The degrees of dissimilarity in animal excitability, in different subjects, may range to the opposite extremes of being fatally and inertly impressed by the same substance; but the shades of resemblance that remotely link, and progressively assimilate the likeness, afford a scale on which may present every intermediate variety. Hence, where most temperamental analogy subsists, will be most uniformity of medicinal operation from any particular agent. This reasoning is not confined to different individuals, but applies with equal force to the same subject, to whom changes of susceptibility are perpetually liable to occur, which might vary, or even contrast, the effect of a former remedy.

What happens hourly must abound, and documents commensurate with this vast source of accumulation croud in proof of the practical fact, *that no invariably curative or specific power, as it has been termed, has ever existed; and in the nature of things, never can.*

The interests of humanity, as well as the just principles and dignity of medical science, imperiously demand a decided distrust in substances so often palmed on public attention for infallible remedies. The laws of animal life, however elementarily similar, are so variously operative in dissimilar constitutions and temperaments, as to render the desideratum of uniform efficiency from an occasionally beneficial medicine absolutely unattainable; rational empiricism will, therefore, be guarded in predicting the accomplishment of cures, though reiterated experience might afford a probable presumption of such an event.

Were it not that inscrutable causes are insensibly generating, and infinitely diversifying the motive powers of animal life, the original simplicity and ordinances of Nature might be steadily manifested, and constantly recognised; every disease would then assume a relative identity of character, and every appropriate remedy would be universally applicable. But, unhappily, this primitive perfection is no longer to be found in the prevailing physical and moral degeneracy of mankind. It is in the motley group of deviations that judicious experience is to search for medicinal efficacy, and to calculate the frequency of definite power.

When a medicinal substance promises to be extraordinarily salutary, it will be of the utmost practical moment to ascertain the precise circumstances in which its curative operation obtains, tains, and the average chance under the ordinary diſſimilarity of conſtitutions for ſimilar effects to occur in different individuals; thus, if the powers of an agent ſhould ſo far tranſcend ſlight diverſities of temperament, as to induce a decided influence on the organic motions of life in a majority of inſtances, it will juſtify a well-founded preſumption in its effects; but yet will not warrant confident reliance. The language and expectation to be held on the power of medicine, ſhould be invariably attempted with doubt, which would ſuitably provide for every contingency.

Argentum nitratum can no more than other relatively active ſubſtances, aſpire to any higher character than that of poſſeſſing *casual power* only in the cure of Epilepſy; therefore, the peculiar temperamental and morbid circumſtances that favour its curative influence, and the probable frequency of their coincidence, conjointly conſtitute the whole of the practical knowledge that either poſſibly can, or need, be required to comprehend the ſcope and energy of its medicinal operation.

I am, &c.

R. KINGLAKE.

*Chilton, super Poldon,*

*April* 3, 1801.

---

