# Errata

**Published:** 1801-06

**Authors:** 


ERRATA.

Page 439, line 30, For "the experience under *this* conſideration," read the experience under conſideration.

Page 441, line 9, For "*attempted* with doubt," read *attempered* with doubt.

END OF VOL. V.

